# Life Satisfaction and Psychological Distress in Relation to Mediterranean Lifestyle Adherence and Behavioural Correlates: Findings from the MEDIET4ALL International Survey

**DOI:** 10.3390/nu18162708

**Published:** 2026-08-19

**Authors:** Achraf Ammar, Atef Salem, Khaled Trabelsi, Martha Montalvan, Bassem Bouaziz, Mohamed Ali Boujelbane, Mohamed Kerkeni, Liwa Masmoudi, Hadeel Ali Ghazzawi, Adam Tawfiq Amawi, Bekir Erhan Orhan, Raynier Zambrano-Villacres, Juliane Heydenreich, Christiana Schallhorn, Tarak Driss, Evelyn Frias-Toral, Piotr Zmijewski, Haitham Jahrami, Waqar Husain, Giuseppe Grosso, Hamdi Chtourou, Wolfgang I. Schöllhorn

**Affiliations:** 1 Department of Training and Movement Science, Institute of Sport Science, Johannes Gutenberg-University Mainz, 55122 Mainz, Germanyschoellw@uni-mainz.de (W.I.S.); 2 Research Laboratory, Molecular Bases of Human Pathology, LR19ES13, Faculty of Medicine of Sfax, University of Sfax, Sfax 3000, Tunisia; 3 Interdisciplinary Laboratory in Neurosciences, Physiology, and Psychology: Physical Activity, Health, and marLearning (LINP2), UFR STAPS, Paris Nanterre University, 92000 Nanterre, France; tdriss@parisnanterre.fr; 4 Department of Nutrition and Food Technology, School of Agriculture, The University of Jordan, Amman 11942, Jordan; h.ghazzawi@ju.edu.jo; 5 High Institute of Sport and Physical Education of Sfax, University of Sfax, Sfax 3000, Tunisia; mohamed.kerkeni@isseps.usf.tn (M.K.); h_chtourou@yahoo.fr (H.C.); 6 Research Laboratory: Education, Motricity, Sport and Health, EM2S, LR19JS01, High Institute of Sport and Physical Education of Sfax, University of Sfax, Sfax 3000, Tunisia; trabelsikhaled@gmail.com (K.T.); liwa.masmoudi@isseps.usf.tn (L.M.); 7 Department of Movement Sciences and Sports Training, School of Sport Science, The University of Jordan, Amman 11942, Jordan; a.amawi@ju.edu.jo; 8 School of Medicine, Universidad Católica de Santiago de Guayaquil, Guayaquil 090615, Ecuador; mmontalvanmd53@gmail.com; 9 Multimedia InfoRmation Systems and Advanced Computing Laboratory (MIRACL), University of Sfax, Sfax 3038, Tunisia; bassem.bouaziz@isims.usf.tn; 10 Higher Institute of Computer Science and Multimedia of Sfax (ISIMS), University of Sfax, Sfax 3038, Tunisia; 11 Faculty of Sports Sciences, Istanbul Aydın University, Istanbul 34295, Türkiye; bekirerhanorhan@aydin.edu.tr; 12 Facultad de Ciencias de la Salud y Desarrollo Humano, Universidad Ecotec, Km. 13.5 Samborondón, Samborondón 092302, Ecuador; razambrano@ecotec.edu.ec; 13 Department of Experimental Sports Nutrition, Faculty of Sports Sciences, Leipzig University, 04103 Leipzig, Germany; juliane.heydenreich@uni-leipzig.de; 14 Department of Sports Economics, Sociology and History, Institute of Sport Science, Johannes Gutenberg-University Mainz, 55131 Mainz, Germany; christiana.schallhorn@uni-mainz.de; 15 Escuela de Medicina, Universidad Espíritu Santo, Samborondón 0901952, Ecuador; evelynft@gmail.com; 16 Department of Biochemistry, Gdansk University of Physical Education and Sport, 80-336 Gdansk, Poland; piotr.zmijewski@insp.waw.pl; 17 Government Hospitals, Manama 329, Bahrain; hjahrami@health.gov.bh; 18 Department of Psychiatry, College of Medicine and Health Sciences, Arabian Gulf University, Manama 329, Bahrain; 19 Department of Humanities, COMSATS University Islamabad, Islamabad 45550, Pakistan; drsukoon@gmail.com; 20 Department of Biomedical and Biotechnological Sciences, University of Catania, 95123 Catania, Italy; giuseppe.grosso@unict.it; 21 Research Unit, Physical Activity, Sport, and Health, UR18JS01, National Observatory of Sport, Tunis 1003, Tunisia

**Keywords:** insomnia severity, life satisfaction, depression, anxiety, stress, social participation, sedentary behaviour, multinational survey, Mediterranean diet

## Abstract

**Background:** Life satisfaction and psychological distress are associated with multiple lifestyle, sleep-related, psychosocial, and sociodemographic factors. However, multinational evidence simultaneously examining these positive and negative psychological dimensions within a comprehensive Mediterranean lifestyle framework remains limited. This study examined correlates of life satisfaction and psychological distress among adults from Mediterranean and neighbouring countries participating in the MEDIET4ALL survey. **Methods:** Data were collected from 4010 adults (59.5% female) across 10 Mediterranean and neighbouring countries using a standardized multilingual e-survey. Life satisfaction was assessed as the primary outcome using the Short Life Satisfaction Questionnaire (SLSQ), whereas psychological distress (depression, anxiety, and stress) was assessed as the secondary outcomes using the Depression Anxiety Stress Scales (DASS-21). Mediterranean lifestyle adherence was assessed using the validated MEDLIFE index, integrating dietary habits with brief indicators of physical activity, rest, and social/convivial habits, while complementary validated measures provided more detailed assessments of physical activity, sedentarily, sleep, insomnia and social participation. Hierarchical multiple linear regression analyses were conducted to identify correlates of the primary and secondary outcomes. Bootstrapped exploratory cross-sectional indirect-association analyses were conducted to examine selected indirect statistical associations involving Mediterranean lifestyle adherence, physical activity, social participation, insomnia severity, life satisfaction, and psychological outcomes. **Results:** Higher life satisfaction was associated with greater Mediterranean lifestyle adherence, better subjective sleep quality, higher social participation, higher education, marital or cohabiting status, and better self-reported health status (β ≈ 0.06–0.18). Conversely, lower life satisfaction was associated with higher body mass index, greater physical activity expenditure, higher insomnia severity, and regional differences between Mediterranean and neighbouring/non-Mediterranean countries (β ≈ −0.05 to −0.19). Insomnia severity showed the strongest negative association with life satisfaction (β = −0.19). Across psychological distress outcomes, insomnia severity emerged as the strongest positive correlate of depression, anxiety, and stress (β ≈ 0.43–0.47), followed by alcohol consumption (β ≈ 0.12–0.15) and residence in Mediterranean countries (β ≈ 0.05–0.08). Older age, analysed as a continuous variable, better self-reported health status, and higher physical activity levels were consistently associated with lower distress levels (β ≈ −0.03 to −0.14). Exploratory cross-sectional indirect-association analyses identified statistically significant indirect associations involving insomnia severity among Mediterranean lifestyle adherence, life satisfaction, and psychological distress. Additional indirect statistical associations involving life satisfaction were observed among physical activity, social participation, and psychological distress outcomes, although their magnitudes were small. **Conclusions:** Mediterranean lifestyle adherence, social participation, physical activity, sleep-related characteristics, and sociodemographic and health-related factors were associated with life satisfaction and psychological distress. Sleep-related variables, particularly insomnia severity and subjective sleep quality, emerged as the most consistent correlates across the psychological outcomes. These cross-sectional findings, including the exploratory indirect statistical associations, should be interpreted as hypothesis-generating and not as evidence of temporal ordering, causal mediation, or behavioural mechanisms.

## 1. Introduction

Mental health disorders remain a major public health challenge worldwide, contributing substantially to disability, reduced quality of life, and growing societal and healthcare burdens [1]. At the same time, mental health is increasingly understood as a multidimensional construct that extends beyond the absence of psychopathology and includes positive dimensions of functioning alongside psychological distress [2,3]. Life satisfaction represents one important positive indicator within this broader perspective and reflects an individual’s global cognitive evaluation of their life circumstances [4]. Importantly, lower life satisfaction has also been prospectively associated with greater subsequent mental-health service use, highlighting its clinical and public-health relevance alongside conventional indicators of psychological distress [5]. Within this broader conception of mental health, increasing attention has been directed toward modifiable lifestyle behaviours that may be associated with both positive psychological outcomes and symptoms of psychological distress, including depression, anxiety, and stress [6,7,8].

The present study was conceptually organized around two complementary frameworks. First, the dual-continuum model of mental health distinguishes positive psychological functioning from psychological distress, indicating that well-being and psychopathological symptoms are related but non-equivalent dimensions rather than opposite ends of a single continuum [2,3]. Accordingly, life satisfaction was examined as the study’s specific positive psychological outcome. Life satisfaction represents the cognitive evaluative component of subjective well-being but does not encompass the broader multidimensional conceptualizations of psychological well-being [9,10]; depression, anxiety, and stress were examined separately as indicators of psychological distress. Second, among the various lifestyle frameworks proposed for health promotion, the Mediterranean lifestyle provides a particularly comprehensive and integrative model because it extends beyond dietary habits to encompass a broader constellation of interrelated behaviours and daily practices that may jointly influence long-term health outcomes. In the present study, this framework was operationalized using the Mediterranean Lifestyle Index (MEDLIFE), which captures three interrelated domains including Mediterranean food consumption, Mediterranean dietary habits, and behaviours related to physical activity, rest, social interaction, and conviviality [11]. This multidimensional formulation reflects the premise that health-related behaviours occur as interconnected patterns within everyday life rather than as isolated habits. The MEDLIFE questionnaire was selected because it is one of the few validated instruments specifically developed to operationalize this broader Mediterranean lifestyle concept by integrating both dietary and non-dietary behavioural domains within a single framework [11,12]. By integrating both dietary and non-dietary lifestyle dimensions, MEDLIFE provides a more comprehensive representation of the traditional Mediterranean way of living than dietary indices alone [11]. This broader perspective is important because lifestyle patterns, rather than isolated behaviours, may better capture the way health-related habits cluster in real-world settings and jointly influence long-term outcomes. Indeed, adherence to the Mediterranean lifestyle has been associated with lower all-cause mortality, and the lifestyle-related components of the MEDLIFE framework appear to contribute importantly beyond dietary habits alone [13]. This distinction may be particularly relevant for mental health research, as accumulating evidence suggests that integrated lifestyle patterns characterised by clusters of healthy behaviours exhibit stronger and more consistent associations with psychological outcomes than isolated behaviours examined separately [6,8,14]. More broadly, the Mediterranean dietary pattern has consistently been associated with favourable health outcomes across multiple domains [15], further supporting the Mediterranean lifestyle as a relevant framework for holistic health promotion. Accordingly, examining life satisfaction alongside psychological distress within a Mediterranean lifestyle framework may provide a more comprehensive and ecologically valid understanding of the behavioural context in which these outcomes are associated. In the present study, MEDLIFE was therefore treated as a self-reported observational adherence score reflecting the behaviours represented by the instrument rather than as an objective or culturally invariant measure of a single homogeneous Mediterranean lifestyle construct across participating countries.

Within this conceptual framework, sleep, physical activity, sedentary behaviour, and social participation were selected as complementary behavioural domains because they represent core components of, or behaviours closely related to, the broader Mediterranean lifestyle and have independently established associations with psychological health. Consistent with this conceptual framework, accumulating evidence supports the relevance of each of these behavioural domains for psychological health. Higher adherence to Mediterranean dietary patterns has been associated with lower depressive risk and more favourable mental health outcomes in both observational and interventional literature [16,17]. Likewise, physical activity is now supported by strong evidence as an effective strategy for improving symptoms of depression, anxiety, and psychological distress [18], and more recent synthesis work indicates that physical activity has also been associated with psychosocial factors relevant to mental health, such as social support, belonging, and life satisfaction [19]. Sedentary behaviour was considered separately because it represents a distinct movement-related exposure that cannot be inferred solely from physical-activity levels and has been independently associated with adverse health and psychological outcomes [20]. Sleep also plays a central role in mental health, with meta-analytic evidence from randomized trials suggesting that improvements in sleep quality are associated with meaningful reductions in depression, anxiety, and stress [21]. In parallel, a recent systematic review reported that greater adherence to the Mediterranean diet is generally associated with better sleep quality and more favourable sleep-related outcomes, including lower insomnia symptoms [22]. Social participation constitutes another important dimension, with growing evidence indicating that lower social participation is associated with a greater likelihood of depressive outcomes, particularly in population-based studies and recent meta-analytic work [23]. Together, these domains provide a theoretically coherent basis for examining psychological health within a multidimensional lifestyle framework. Nevertheless, because all variables were measured cross-sectionally, the present framework organizes the examined associations without specifying temporal ordering or causal mechanisms.

Despite this growing evidence, several gaps remain within the existing literature. First, research examining Mediterranean patterns and mental health has predominantly focused on Mediterranean diet adherence rather than the broader Mediterranean lifestyle construct encompassing dietary, movement-related, rest, and social dimensions [11,13]. Second, sleep, physical activity, sedentary behaviour, and social participation have frequently been investigated as isolated behaviours, limiting understanding of how these domains jointly relate to psychological health within an integrated lifestyle framework [6,7]. Third, studies have more often focused on negative psychological outcomes such as depression and anxiety than on positive dimensions of mental health, although life satisfaction and psychological distress represent distinct but complementary constructs [2,24]. Finally, much of the available evidence derives from single-country studies or selected population groups, limiting understanding of how lifestyle–mental health relationships operate across diverse sociocultural settings, while multinational investigations using a common multilingual protocol remain relatively scarce [6,22,25,26]. Consequently, simultaneously examining these predefined lifestyle domains within a multivariable framework may provide a more comprehensive understanding of their independent and mutually adjusted associations with positive and negative dimensions of mental health, while recognising that cross-sectional analyses cannot establish temporal or causal relationships.

Addressing these limitations requires large-scale datasets capable of simultaneously capturing lifestyle, sleep, psychosocial, and mental health dimensions across diverse sociocultural settings. The MEDIET4ALL project offers a timely opportunity to address this need [27]. Using a standardized multilingual international e-survey administered across ten Mediterranean and neighbouring countries, the project was designed to assess Mediterranean lifestyle adherence together with a broad range of behavioural, psychosocial, and sociodemographic correlates [28,29,30,31,32,33]. Previous MEDIET4ALL analyses have characterised heterogeneity in Mediterranean lifestyle adherence and related behaviours according to sex [28], region [29], and country [33], as well as significant associations between adherence and variables such as physical activity, sleep-related indicators, and psychosocial measures [30]. Complementary analyses further examined self-reported health status [30] and physical activity and sedentary behaviour [34] as the primary outcomes, respectively, while Mediterranean lifestyle adherence and related behavioural and psychosocial variables were considered explanatory factors. Although psychological variables were included in some of these previous studies as covariates, explanatory variables, or correlates, they were not jointly examined as the principal psychological outcomes within a common theoretical and analytical framework.

The present study instead addresses a distinct psychological-health-focused research question by treating life satisfaction as the primary outcome and depression, anxiety, and stress as secondary psychological outcomes within a common hierarchical multivariable framework. Rather than reproducing previously published primary outcome analyses, it simultaneously evaluates the independent and mutually adjusted associations of Mediterranean lifestyle adherence, movement behaviours, sleep characteristics, social participation, and predefined sociodemographic and health-related factors with both life satisfaction and psychological distress. This analytical approach helps fill an important gap in both the broader literature and the MEDIET4ALL research programme, where psychological variables have generally been considered explanatory or descriptive variables rather than outcomes of primary interest, and where relatively few multinational studies have jointly examined positive psychological well-being and psychological distress within a comprehensive Mediterranean lifestyle framework. Against this background, the primary objective of this secondary analysis of the MEDIET4ALL survey was to examine the association between Mediterranean lifestyle adherence and life satisfaction in the multinational analytical sample after progressive adjustment for predefined sociodemographic, health-related, movement-related, sleep-related, and social variables. Secondary objectives were to identify the independent correlates of depression, anxiety, and stress, evaluate the robustness of the principal associations across sex and the broad Mediterranean versus neighbouring/non-Mediterranean comparison-country grouping, and explore selected cross-sectional indirect associations involving insomnia severity and life satisfaction.

These exploratory analyses were motivated by previous theoretical and empirical evidence indicating that Mediterranean lifestyle adherence is associated with more favourable sleep characteristics, while sleep health is consistently associated with both psychological well-being and psychological distress [21,22,35]. Likewise, physical activity and social participation have consistently been associated with greater life satisfaction and subjective well-being, whereas life satisfaction itself has been associated with lower levels of depression, anxiety, and stress [19,23]. Accordingly, insomnia severity and life satisfaction were considered theoretically relevant variables for exploratory cross-sectional indirect-association analyses. These analyses were intended to generate hypotheses regarding the statistical structure of the observed associations and to inform future longitudinal and intervention research. They were not designed to establish temporal ordering, causal direction, or mediation.

It was hypothesised that stronger Mediterranean lifestyle adherence, greater physical activity, higher social participation, healthier sleep characteristics, and lower sedentary behaviour would be associated with higher life satisfaction and lower depression, anxiety, and stress scores. Furthermore, based on previous theoretical and empirical evidence [19,21,22], we hypothesized, on an exploratory and explicitly non-causal basis, that statistically significant cross-sectional indirect associations involving insomnia severity and life satisfaction would be observed among selected lifestyle factors and psychological outcomes.

## 2. Materials and Methods

### 2.1. Study Design and Participants

This secondary analysis draws on the multinational MEDIET4ALL survey, which investigated Mediterranean lifestyle adherence and a broad range of behavioural, sociodemographic health-related, and psychosocial characteristics, in adults from Mediterranean and neighbouring/non-Mediterranean countries [28,29,30,31,32,33]. The survey was implemented in Germany, Luxembourg, Italy, France, Spain, Algeria, Tunisia, Morocco, Jordan, and Türkiye.

Data collection took place over approximately four months beginning in summer 2024. The questionnaire was prepared collaboratively by a multidisciplinary consortium with expertise spanning nutrition and public health, psychology, behavioural and social sciences, nutrition, and sport and movement science. A common electronic survey was offered in English, German, Italian, French, Spanish, Turkish, and Arabic, and to support participation across the ten study countries. Survey administration was conducted using SoSci Survey on infrastructure hosted by Johannes Gutenberg University Mainz, Germany, in accordance with applicable GDPR requirements.

Recruitment relied on an open, non-probability strategy combining convenience, network-based, and participant-supported dissemination. The study invitation was circulated through participating institutions and consortium partners, university and institutional webpages, professional and academic mailing networks, direct electronic communication, and social-media channels including Facebook, LinkedIn, ResearchGate, X/Twitter, and WhatsApp. Investigators and collaborating institutions also distributed the invitation within their respective national networks, while recipients were free to share the survey further. This approach facilitated recruitment across geographically and culturally diverse settings but was not designed to generate nationally representative samples. No population sampling frames, probability-selection procedures, or country-level recruitment quotas were used; consequently, the resulting sample should be interpreted as a multinational analytical cohort rather than as representative of the adult populations of the participating countries.

Individuals could participate if they were at least 18 years old, lived in one of the participating countries, were able to complete one of the available language versions, and provided electronic informed consent.

For the present analysis, participants were additionally required to meet the analytical completeness requirements by having complete data for the psychological outcomes and all sociodemographic, health-related, behavioural, sleep-related, and psychosocial variables included in the regression models. Accordingly, the analyses followed a complete-case approach, whereby participants with missing values for any variable required in the corresponding regression models were excluded from the analytical dataset, and no statistical imputation was performed. Responses were excluded if they represented partial submissions, lacked electronic informed consent, contained missing data for variables required in the present analyses, showed internally inconsistent response patterns, were identified as duplicate or near-duplicate entries, or included implausible anthropometric, behavioural, or sleep-related values. These quality-control procedures were applied iteratively, and the exclusion criteria were not mutually exclusive; consequently, a single survey entry could satisfy more than one exclusion criterion. Duplicate or near-duplicate entries were screened using IP address information, response timestamps, highly similar demographic characteristics, and response patterns. Logic-based consistency checks were also applied across related items, for example to identify contradictory physical activity or sleep responses. Implausible values, such as unrealistic anthropometric values or impossible sleep durations, were removed during data cleaning. Because many excluded entries represented incomplete submissions, duplicate or near-duplicate responses, internally inconsistent records, or implausible values, they were treated as incomplete or invalid survey records and were not assumed to contain data missing at random for the purpose of statistical imputation. Overall, the survey initially received more than 8000 entries. Following the structured quality-control procedures, including assessment of eligibility, questionnaire completeness, duplicate or near-duplicate records, internal consistency, and plausibility of anthropometric, behavioural, and sleep-related data, 4010 valid and complete responses, representing approximately one half of the recorded survey entries, were retained for the final analytical sample.

For the regional variable, countries were grouped according to the regional classification used in previous MEDIET4ALL analyses. France, Italy, Spain, Tunisia, Algeria, Morocco, and Türkiye were classified as Mediterranean countries, whereas Germany, Luxembourg, and Jordan were classified as neighbouring/non-Mediterranean comparison countries [28]. This grouping was used only as a broad contextual variable for regional comparison and should not be interpreted as implying cultural, behavioural, socioeconomic, or lifestyle homogeneity within regions.

Ethical approval was granted by the Ethics Committee of the Faculty of Medicine, University of Sfax (approval 058/24), and study procedures were conducted in accordance with the Declaration of Helsinki. Participation was voluntary and anonymous. No directly identifying information was collected, and respondents could discontinue the questionnaire before final submission. Electronic submission of the completed questionnaire constituted consent for the anonymous research use of the supplied data.

### 2.2. Survey Instruments and Measured Variables

The MEDIET4ALL survey was designed to capture multiple dimensions of the Mediterranean lifestyle, as well as psychosocial variables, sociodemographic characteristics, and health behaviours. The survey incorporated validated instruments and standardized self-report measures commonly used in epidemiological research. All major questionnaires used in the survey have previously demonstrated acceptable reliability and validity in their original validation studies and/or official translated versions. To facilitate multinational implementation, the MEDIET4ALL questionnaire was administered in seven languages (English, French, German, Spanish, Italian, Turkish, and Arabic). For instruments or items lacking validated versions in some languages, forward–backward translation procedures were applied following established guidelines of cross-cultural adaptation. Prior to dissemination, the multilingual survey underwent internal pilot testing. Test–retest reliability analyses conducted during the pilot phase demonstrated good-to-excellent stability across translated items, with correlation coefficients ranging from r = 0.81 to 0.94 [29,30]. These procedures supported harmonized multilingual survey administration and the reliability of the translated questionnaires but were not intended to establish formal cross-cultural measurement invariance across countries or language versions. Reported psychometric properties reflect both previously validated instruments and internal quality-control procedures conducted within the MEDIET4ALL project.

### 2.3. Psychological Outcomes

Life satisfaction, the primary psychological outcome, was measured using the Short Life Satisfaction Questionnaire (SLSQ). This three-item measure is based on the conceptual approach of the Satisfaction with Life Scale developed by Diener and colleagues [4]. Each item is answered on a seven-point response scale ranging from 1 (“strongly disagree”) to 7 (“strongly agree”). Item responses are summed to yield a total score between 3 and 21, with larger values reflecting greater satisfaction with life. The underlying Satisfaction with Life Scale framework has shown strong internal consistency and temporal stability across different populations [4]. The shortened measure has also been used in previous large-scale population-based research, including studies conducted during the COVID-19 confinement period, as well as in earlier MEDIET4ALL analyses [28,29,30,32,33].

Psychological distress was evaluated with the 21-item Depression Anxiety Stress Scales (DASS-21) [36]. The questionnaire comprises three seven-item subscales assessing depressive symptoms, anxiety symptoms, and stress-related experiences. Participants indicate how closely each statement applied to them using four response options scored from 0 (“did not apply to me at all”) to 3 (“applied to me very much or most of the time”). Scores were derived separately for the depression, anxiety, and stress domains according to the recommended scoring procedure, with higher subscale values indicating greater symptom burden. The DASS-21 has been extensively evaluated and has shown strong internal consistency and construct validity across different populations and translated versions [36,37].

### 2.4. Mediterranean Lifestyle Framework and Related Behavioural Domains

The Mediterranean lifestyle was operationalized primarily through the MEDLIFE adherence score, supplemented by separate domain-specific assessments of movement behaviours, sleep, and social participation. MEDLIFE provides an overall multidimensional adherence index incorporating extensive dietary content together with brief indicators related to physical activity, rest, social habits, and conviviality. To characterize these behavioural domains in greater detail and examine their independent associations with the psychological outcomes after accounting for overall MEDLIFE adherence, physical activity and sedentarily, sleep characteristics and insomnia, and social participation were additionally assessed using dedicated instruments, as described below.

#### 2.4.1. Mediterranean Lifestyle Adherence (MEDLIFE)

The Mediterranean Lifestyle Index (MEDLIFE) was used to quantify self-reported adherence to behaviours traditionally associated with the Mediterranean lifestyle [11]. The instrument covers three broad components: Mediterranean food-consumption patterns; dietary practices and habits; and a combined domain addressing physical activity, rest, social habits, and conviviality. Scoring followed the published MEDLIFE procedure, whereby higher values indicate greater adherence [11,31]. The instrument has previously shown acceptable psychometric performance in population-based research [11].

For the present multinational analysis, the MEDLIFE total score was used as an observational indicator of reported adherence to the dietary and non-dietary behaviours represented by the instrument. It was not assumed to constitute an objective measure or a culturally invariant representation of a single homogeneous Mediterranean lifestyle across participating countries and language versions.

#### 2.4.2. Physical Activity and Sedentary Behaviour

Movement behaviour was assessed using the short form of the International Physical Activity Questionnaire (IPAQ-SF) [38]. Weekly physical-activity volume was expressed as metabolic-equivalent minutes per week (MET-min/week), incorporating reported walking and moderate- and vigorous-intensity activity. Participants additionally reported the time they typically spent sitting each day, which was analysed as a separate sedentary-behaviour indicator. The IPAQ-SF has been extensively used in international epidemiological research and has demonstrated acceptable measurement properties, although, as with other self-reported activity instruments, its estimates remain susceptible to recall and reporting error [38].

#### 2.4.3. Sleep Characteristics and Insomnia

Sleep was represented by several complementary indicators rather than by a single summary variable. Selected questions adapted from established sleep instruments, including the Pittsburgh Sleep Quality Index framework, provided information on habitual sleep duration, time required to fall asleep, sleep efficiency, and subjective sleep quality [28,29,32,33]. Sleep efficiency was derived as the percentage of time in bed hat was reported as actual sleep:

Sleep efficiency (%) = (actual sleep duration/time spent in bed) × 100.

Participants rated their overall sleep quality on four ordered response levels, coded from 1 (“very bad”) to 4 (“very good”), such that larger values represented more favourable perceived sleep quality.

Insomnia-related symptoms were assessed separately with the seven-item Insomnia Severity Index (ISI) [39]. The ISI captures difficulties initiating and maintaining sleep, early-morning awakening, dissatisfaction with sleep, and perceived consequences of sleep difficulties. Items are scored from 0 to 4 and summed to produce a total ranging from 0 to 28, with larger totals indicating greater insomnia severity. Interpretation followed established ISI severity categories. The instrument has demonstrated satisfactory internal consistency, temporal stability, and convergent validity in both clinical and community samples [39].

#### 2.4.4. Social Participation and Technology Use

Social participation was measured using the Short Social Participation Questionnaire (SSPQ). Its 14 items assess participation in social activities and interpersonal interactions; ten items use graded response options and four use dichotomous responses. Item responses are combined into an overall score ranging from 14 to 70, with higher values representing greater social participation.

Technology-related lifestyle use was characterized with the Short Technology-Use Questionnaire (STuQL). Three items assess how frequently technology is used in connection with social participation, dietary practices, and physical activity. Responses range from “never” to “all the time” on a five-point scale and yield a total score between 3 and 15, with higher totals reflecting more frequent technology use in these lifestyle contexts. Both measures have been used previously in related lifestyle research [30,31,32,33,40].

### 2.5. Sociodemographic, Health-Related, and Contextual Covariates

The survey collected sex, age, country of residence, educational attainment, employment and marital status, and type of residential environment (rural, suburban, or urban). Additional health and lifestyle information comprised body weight and height, alcohol use, smoking, and perceived health status. Age was retained as a continuous variable in the regression analyses. Body mass index was derived from reported weight and height as kg/m^2^. For descriptive interpretation, BMI values were considered according to World Health Organization categories:<18.5 kg/m^2^, 18.5–24.9 kg/m^2^, 25.0–29.9 kg/m^2^, and ≥30.0 kg/m^2^ for underweight, normal weight, overweight, and obesity, respectively. Because both height and weight were self-reported, BMI was regarded as an estimated rather than objectively measured anthropometric indicator [28,29,30,32].

Self-reported health was classified as healthy, at increased risk of disease, or currently affected by a diagnosed disease. Consistent with previous MEDIET4ALL analyses, the at-risk classification included respondents reporting combinations of two or more cardiovascular risk characteristics, such as hypertension, dyslipidaemia, overweight/obesity, smoking, or physical inactivity, and/or relevant histories including cardiovascular disease or diabetes, or combinations thereof [28,29,30,33].

These sociodemographic, health-related, and contextual covariates were selected a priori on the basis of existing literature and data availability, considered potential confounders, and included in the hierarchical regression models where appropriate.

### 2.6. Statistical Analysis

Analyses were undertaken in IBM SPSS Statistics, version 29 (Chicago, IL, USA). Sample characteristics were summarized using means with standard deviations for continuous measures and counts with percentages for categorical measures.

Hierarchical multiple linear regression was used to evaluate variables associated with the psychological outcomes. The SLSQ total score served as the primary outcome, whereas depression, anxiety, and stress scores from the DASS-21 were examined separately as secondary outcomes. The SLSQ total score and DASS-21 subscale scores were analysed as continuous summed multi-item outcomes because the study aimed to estimate adjusted mean-score differences across predictors. Multiple linear regression also permitted direct comparison of standardized coefficients and evaluation of the incremental explained variance contributed by the successive hierarchical predictor blocks. The suitability of this approach was evaluated from the behaviour and approximate distribution of the model residuals rather than from a requirement that the raw outcome scores themselves be normally distributed. In all analyses, predictors were entered sequentially in conceptually defined blocks following a hierarchical modelling strategy. This approach allowed stepwise adjustment for sociodemographic and health-related covariates while also examining the incremental contribution of each predictor block to the explained variance of psychological outcomes.

Model 1 comprised the sociodemographic block, including age, sex, country or region, education level, employment status, marital status, and living environment. Model 2 retained these covariates and incorporated an additional health-related block consisting of body mass index (BMI), smoking status, alcohol consumption, and self-reported health status. Subsequent models incorporated behavioural and lifestyle variables. Model 3 added Mediterranean lifestyle adherence (Block 3) using the MEDLIFE total score. The domain-specific behavioural measures were entered in subsequent model blocks while retaining the MEDLIFE total score, allowing their independent associations with the psychological outcomes to be examined after accounting for overall Mediterranean lifestyle adherence. Specifically, Model 4 further included physical activity and sedentary behaviour indicators (Block 4), namely weekly physical activity energy expenditure (MET-minutes per week) and daily sitting time. Model 5 further incorporated sleep-related measures (Block 5), comprising sleep duration, sleep latency, sleep efficiency, subjective sleep quality, and Insomnia Severity Index scores. Model 6 subsequently introduced social participation and technology-use measures as the final predictor domain. Age was modelled continuously throughout all regression analyses; consequently, its regression coefficient represents the estimated change in the outcome associated with each one-year increase in age rather than differences between predefined age groups. This sequential modelling framework allowed examination of the associations between behavioural and lifestyle factors and psychological outcomes after progressive adjustment for sociodemographic and health-related covariates. Because the present study was not designed or powered to estimate country-specific slopes or multilevel random-slope models across only ten country-level units with unequal sample sizes, pooled regression models were used to examine overall associations, while country/region-related contextual variables were included to account for broad contextual differences. Accordingly, the analyses were intended to identify overall correlates within the multinational analytical sample rather than to estimate country-specific effects. The fully adjusted Model 6 was designated as the principal inferential model because it retained all 21 conceptually prespecified sociodemographic, health-related, movement-related, sleep-related, and psychosocial predictors. With 4010 complete observations, this model included approximately 191 observations per estimated predictor, thereby substantially reducing concern about conventional parameter-to-sample-size overfitting. Model 7 was subsequently derived as a parsimonious descriptive summary to facilitate presentation of the principal findings by retaining predictors that remained statistically significant in Model 6. Because this data-driven variable-retention procedure may yield optimistic coefficients and *p*-values, inferential interpretation was based primarily on the fully adjusted Model 6.

Sensitivity analyses were performed to assess whether the primary association between Mediterranean lifestyle adherence and life satisfaction was consistent across sex and between Mediterranean and neighbouring/non-Mediterranean regional groups. The fully adjusted regression model (Model 6) was estimated separately within each subgroup while retaining all covariates included in the primary analysis, except the corresponding stratification variable. In addition, formal interaction analyses were performed by introducing MEDLIFE × region and MEDLIFE × sex interaction terms into the corresponding fully adjusted pooled regression models. MEDLIFE scores were mean-centred before construction of the interaction terms. Differences between subgroups were evaluated primarily from the interaction terms and their corresponding 95% confidence intervals rather than from differences in subgroup-specific statistical significance.

To evaluate the robustness of nominal statistical significance to multiple testing, the Benjamini–Hochberg false-discovery-rate (FDR) procedure was applied separately to the predictor tests within each fully adjusted psychological-outcome model. Primary and secondary outcomes were treated as separate multiplicity families. Nominal *p*-values and FDR-adjusted q-values are reported in Supplementary Table S7. Interpretation emphasized effect estimates, standardized regression coefficients, 95% confidence intervals, explained variance, consistency across related outcomes, and robustness after FDR correction rather than nominal statistical significance alone.

Exploratory bootstrapped cross-sectional indirect-association analyses were also conducted to quantify selected indirect statistical associations among Mediterranean lifestyle adherence, physical activity, social participation, insomnia severity, life satisfaction, and psychological outcomes. Based on theoretical considerations, prior literature, and previous MEDIET4ALL findings, three restricted conceptual models were evaluated: (i) a model involving Mediterranean lifestyle adherence, insomnia severity, and life satisfaction; (ii) models involving physical activity, life satisfaction, and depression, anxiety, or stress; and (iii) models involving social participation, life satisfaction, and psychological distress. These directionally specified models represented one theoretically informed statistical ordering of the variables but were not assumed to establish temporal precedence or causal direction. In the model estimation, Mediterranean lifestyle adherence, physical activity, and social participation were entered as predictor variables; insomnia severity or life satisfaction represented the variables involved in the indirect statistical association; and life satisfaction or the DASS-21 subscale scores were entered as outcome variables. All models were adjusted for age, sex, region of residence, education level, employment status, body mass index, smoking status, alcohol consumption, and self-reported health status.

Indirect associations were quantified using 5000 bootstrap resamples, from which 95% confidence intervals (CIs) were derived. Statistical evidence for an indirect association was considered present when the respective bootstrapped CI excluded zero (Preacher & Hayes, 2008 [41]; Hayes, 2018 [42]). Because all variables were assessed cross-sectionally, these prespecified and theoretically restricted analyses were regarded exclusively as exploratory and hypothesis-generating. Their results were therefore not considered evidence of causal mediation, temporal precedence, or directionality, nor were conclusions based solely on nominal statistical significance.

Model assumptions and diagnostics were examined before interpreting the regression estimates. Linearity and homoscedasticity were evaluated by inspecting plots of residuals against fitted values, while residual histograms and normal Q–Q plots were used to assess the approximate residual distribution. Potential multicollinearity was examined through tolerance statistics and variance inflation factors (VIF), with tolerance values >0.20 and VIF values <5 considered to indicate the absence of problematic collinearity [43]. Diagnostic results for the fully adjusted model are reported in the Supplementary Material.

This secondary analysis was not prospectively preregistered. Nevertheless, the designation of the primary outcome, organization of predictors into hierarchical conceptual blocks, and principal study hypotheses were determined from the underlying study framework and existing literature before the final analyses were completed. In contrast, the cross-sectional indirect-association analyses were explicitly designated as exploratory and were interpreted accordingly as hypothesis-generating. All inferential tests were two-sided, with the statistical significance threshold set at *p* < 0.05.

## 3. Results

### 3.1. Descriptive Characteristics of the Study Population

The final analytical sample included 4010 participants from ten countries. Overall, 2170 participants (54.1%) were aged 18–35 years, 1171 (29.2%) were aged 36–55 years, and 669 (16.7%) were older than 55 years. The sample comprised 2385 women (59.5%) and 1625 men (40.5%). Of the total sample, 2594 participants (64.7%) resided in Mediterranean countries and 1416 (35.3%) in neighbouring/non-Mediterranean comparison countries. Country participation was as follows: Algeria (*n* = 146, 3.6%), France (*n* = 533, 13.3%), Germany (*n* = 616, 15.4%), Italy (*n* = 711, 17.7%), Luxembourg (*n* = 118, 2.9%), Tunisia (*n* = 170, 4.2%), Spain (*n* = 278, 6.9%), Morocco (*n* = 160, 4.0%), Türkiye (*n* = 596, 14.9%), and Jordan (*n* = 682, 17.0%). Because recruitment relied on online convenience and network-based dissemination, these distributions reflect sample composition rather than population representativeness within participating countries. Detailed descriptions of the MEDIET4ALL sample characteristics across regions, sex and adherence categories have been reported in previous publications [28,29]. Table 1 provides a summary of the analytical sample used in the present analyses.

### 3.2. Hierarchical Regression Analyses Examining Correlates of Life Satisfaction

Hierarchical multiple linear regression analyses examining life satisfaction are presented in Table 2. Across the sequential models, the proportion of explained variance increased progressively from 1.9% in Model 1 to 17.2% in Model 6 and 16.8% in the final parsimonious model (Model 7), with the largest incremental improvement observed after the inclusion of sleep-related variables (ΔR^2^ = 0.066), followed by the addition of health-related variables (ΔR^2^ = 0.029), Mediterranean lifestyle adherence (ΔR^2^ = 0.027), and social participation and technology-use indicators (ΔR^2^ = 0.026). Thus, the fully adjusted model explained approximately one-sixth of the variability in life satisfaction, indicating that about 82.8% of the interindividual variance remained unexplained by the variables included in the present analyses.

In the auxiliary parsimonious model (model 7), higher life satisfaction was positively associated after adjustment with higher educational level (B = 0.212, 95% CI: 0.112 to 0.312), being married or cohabiting (B = 0.609, 95% CI: 0.392 to 0.826), better self-reported health status (B = 0.611, 95% CI: 0.385 to 0.838), greater Mediterranean lifestyle adherence (B = 0.129, 95% CI: 0.086 to 0.172), better subjective sleep quality (B = 0.598, 95% CI: 0.398 to 0.797), and higher levels of social participation (B = 0.079, 95% CI: 0.065 to 0.093). Conversely, lower life satisfaction was associated after adjustment with higher body mass index (B = −0.043, 95% CI: −0.071 to −0.015), greater physical activity expenditure (B = −0.074, 95% CI: −0.116 to −0.031), higher insomnia severity (B = −0.149, 95% CI: −0.175 to −0.122), and residing in Mediterranean compared with non-Mediterranean countries (B = −0.478, 95% CI: −0.752 to −0.205).

Among the retained predictors, insomnia severity showed the strongest negative standardized association (β = −0.192), whereas social participation (β = 0.176), subjective sleep quality (β = 0.104), and Mediterranean lifestyle adherence (β = 0.090) showed the strongest positive standardized associations with life satisfaction. Variables such as age, sex, employment status, smoking, alcohol consumption, sedentary behaviour, sleep duration, sleep latency, sleep efficiency, and technology use were not retained in the final model.

Collinearity diagnostics for the fully adjusted model are reported in Supplementary Table S4. Most predictors showed acceptable VIF values, whereas sleep latency and sleep efficiency showed elevated VIFs, indicating potential collinearity among sleep-related indicators. Accordingly, individual coefficients for these sleep variables should be interpreted cautiously, particularly in the fully adjusted model.

Benjamini–Hochberg false-discovery-rate correction confirmed the robustness of the principal life-satisfaction findings. All 10 predictors showing nominal statistical significance in the fully adjusted Model 6 remained significant after FDR correction, including the primary positive association between Mediterranean lifestyle adherence and life satisfaction. The corresponding nominal and FDR-adjusted results for all four psychological outcomes are presented in Supplementary Table S7.

Sensitivity analyses demonstrated that the primary association between Mediterranean lifestyle adherence and life satisfaction was robust across both Mediterranean and neighbouring/non-Mediterranean regions and across sex (Supplementary Tables S5 and S6, respectively). Higher MEDLIFE adherence remained significantly associated with greater life satisfaction in both regional groups and in both men and women. The MEDLIFE × region interaction reached statistical significance, indicating that the positive association was stronger among participants from neighbouring/non-Mediterranean countries, whereas no significant MEDLIFE × sex interaction was observed, supporting the consistency of the primary findings across men and women.

### 3.3. Psychological Distress: Fully Adjusted Analyses and Auxiliary Parsimonious Summaries

Primary inference for depression, anxiety, and stress was based on the fully adjusted Model 6 results provided in Supplementary Tables S1–S3. Table 3 presents the corresponding auxiliary parsimonious Model 7 summaries to facilitate interpretation. Overall, the models explained a moderate proportion of variance in psychological distress outcomes, with adjusted R^2^ values of 0.272 for depression, 0.294 for anxiety, and 0.262 for stress. Across all three outcomes, higher insomnia severity showed the strongest positive association with psychological distress (β = 0.43 to 0.47; all *p* < 0.001), followed by alcohol consumption (β = 0.12 to 0.15; all *p* < 0.001) and residence in Mediterranean countries (β = 0.05 to 0.08; all *p* < 0.001). Conversely, older age (β = −0.12 to −0.14; all *p* < 0.001), better self-reported health status (β = −0.07 to −0.10; all *p* < 0.001), and higher physical activity levels (β = −0.03 to −0.08; all *p* < 0.001) were consistently associated with lower depression, anxiety, and stress scores after adjustment.

Additional associations were outcome-specific. For depression, lower distress scores were observed among married or cohabiting participants (β = −0.04, *p* = 0.010), participants with higher educational attainment (β = −0.04, *p* < 0.001), and those with higher levels of social participation (β = −0.06, *p* < 0.001), whereas greater sedentary behaviour (β = 0.04, *p* = 0.001) was associated with higher depressive symptoms. For anxiety, higher scores were additionally associated with female gender (β = 0.04, *p* = 0.008), lower educational level (β = −0.05, *p* < 0.001), greater difficulty falling asleep (β = 0.11, *p* < 0.001), and longer sleep duration (β = 0.05, *p* < 0.001). For stress, female gender (β = 0.08, *p* < 0.001) and sedentary behaviour (β = 0.04, *p* = 0.007) were positively associated with higher stress scores.

After Benjamini–Hochberg correction, all 11 nominally significant predictors in the fully adjusted depression model remained statistically significant. In the anxiety model, 11 of the 12 nominally significant predictors remained significant, whereas the association with sleep duration did not survive correction (q = 0.0643). In the stress model, eight of the 10 nominally significant predictors remained significant, whereas employment status (q = 0.1039) and subjective sleep quality (q = 0.0842) did not remain significant after correction. Accordingly, these three associations are interpreted as nominal and exploratory rather than robust findings.

Overall, insomnia severity showed the strongest positive association with all three psychological distress dimensions, while several sociodemographic, lifestyle, sleep-related, and psychosocial variables displayed outcome-specific associations.

### 3.4. Exploratory Bootstrapped Cross-Sectional Indirect-Association Analyses of Lifestyle Factors and Psychological Outcomes

**Figure 1:**
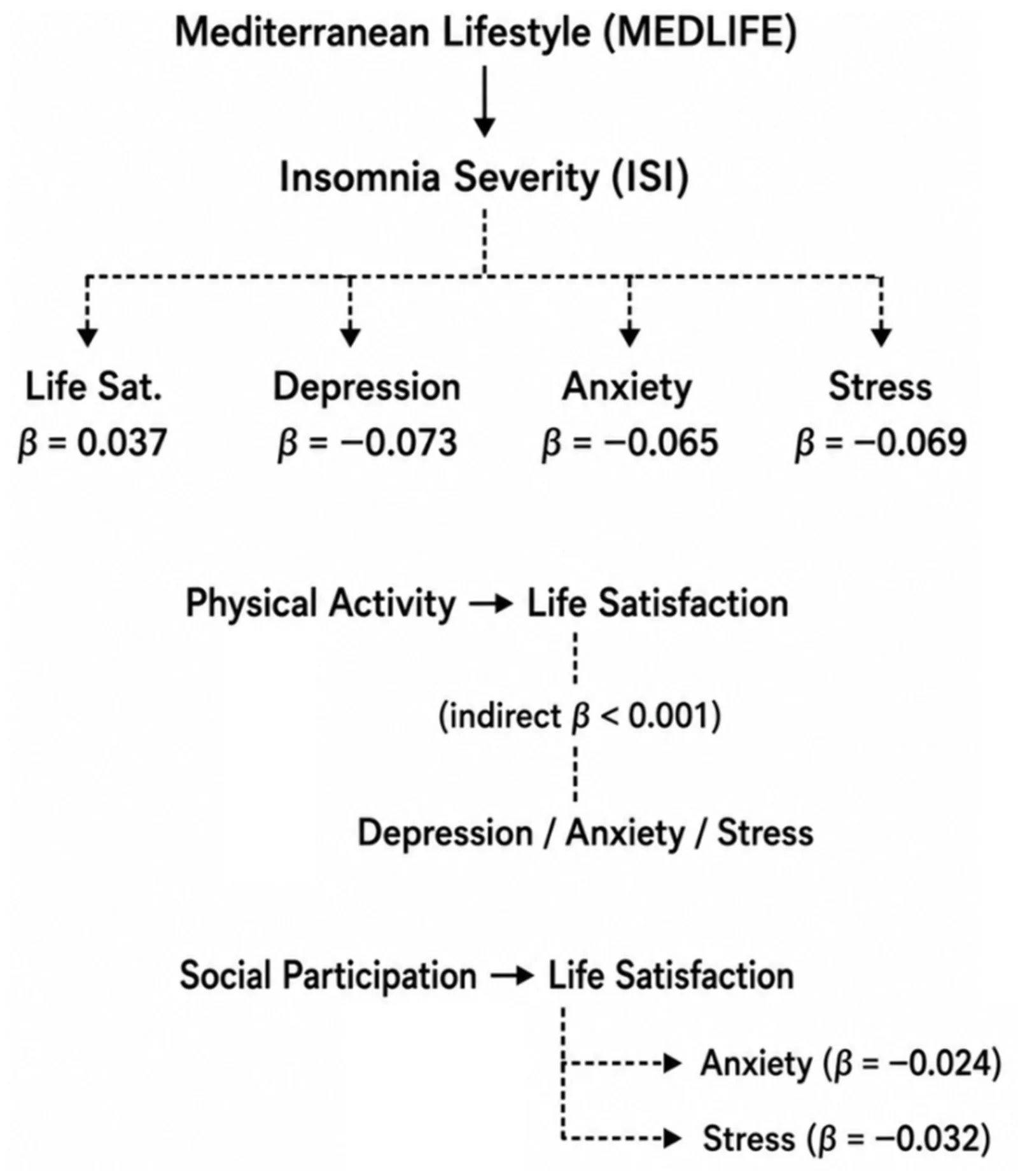
Conceptual representation of the exploratory cross-sectional indirect statistical associations involving Mediterranean lifestyle adherence, behavioural factors, and psychological outcomes (indirect β). Note: Arrows indicate the statistical ordering specified for the exploratory analyses and should not be interpreted as evidence of temporal sequence, causal direction, or behavioural mechanisms.

Results of the exploratory bootstrapped cross-sectional indirect-association analyses are presented in Table 4 and Figure 1. Within the Mediterranean lifestyle–insomnia conceptual model, indirect statistical associations involving insomnia severity were observed between Mediterranean lifestyle adherence and life satisfaction(indirect β = 0.0374, 95% CI: 0.0262 to 0.0496), depression (indirect β = −0.0727, 95% CI: −0.0943 to −0.0528), anxiety (indirect β = −0.0650, 95% CI: −0.0838 to −0.0465), and stress (indirect β = −0.0688, 95% CI: −0.0895 to −0.0495). Within the physical activity–life satisfaction conceptual model, indirect statistical associations involving life satisfaction were observed between physical activity and depression, anxiety, and stress, although the magnitude of the observed indirect associations was very small. Within the social participation–life satisfaction conceptual model, indirect statistical associations involving life satisfaction were observed between social participation and anxiety (indirect β = −0.0240, 95% CI: −0.0288 to −0.0193) and stress (indirect β = −0.0315, 95% CI: −0.0375 to −0.0259).

## 4. Discussion

The present study examined the behavioural, sociodemographic, sleep-related, and psychosocial correlates of life satisfaction and psychological distress in a multinational sample from Mediterranean and neighbouring countries. Within the multidimensional Mediterranean lifestyle framework applied in this study, overall lifestyle adherence was operationalized using the self-reported MEDLIFE score, while movement behaviours, sleep characteristics, and social participation were assessed using complementary, more detailed domain-specific measures. Overall, the regression analyses indicated that life satisfaction and psychological distress were associated with a multidimensional set of lifestyle and health-related factors. Consistent with the primary objective and hypothesis, Mediterranean lifestyle adherence remained positively associated with life satisfaction after adjustment for sociodemographic, health-related, movement-related, sleep-related, and social variables. Higher life satisfaction was also associated with better subjective sleep quality, greater social participation, higher educational attainment, and better self-reported health status, whereas lower life satisfaction was associated with higher body mass index, greater insomnia severity, higher physical activity expenditure, and regional differences between Mediterranean and neighbouring/non-Mediterranean countries.

Beyond life satisfaction, higher physical activity, older age, and better self-reported health status were consistently associated with lower psychological distress, whereas insomnia severity emerged as the strongest positive correlate across all three distress outcomes. The exploratory cross-sectional indirect-association analyses further identified selected indirect statistical associations involving insomnia severity and life satisfaction that were consistent with the broader pattern of the regression findings.

Taken together, these findings highlight the importance of considering life satisfaction alongside psychological distress within a broader lifestyle framework encompassing Mediterranean lifestyle adherence, movement behaviours, sleep health, and social participation. The principal findings also proved robust to both false-discovery-rate correction and the prespecified region- and sex-specific sensitivity analyses, supporting the stability of the principal associations observed within this multinational analytical sample. However, given the cross-sectional design, all observed associations, including the exploratory indirect associations, should be interpreted cautiously and not as evidence of causal relationships, behavioural mechanisms, or temporal pathways.

### 4.1. Mediterranean Lifestyle Adherence and Life Satisfaction

Consistent with the primary objective and hypothesis, Mediterranean lifestyle adherence was positively associated with life satisfaction after adjustment for sociodemographic, health-related, movement-related, sleep-related, and social variables. This finding supports the broader Mediterranean lifestyle concept rather than a diet-only interpretation and is consistent with international evidence suggesting that Mediterranean dietary and lifestyle patterns are linked to more favourable mental health profiles [6,16,17]. These findings were further supported by sensitivity analyses demonstrating that the positive association remained evident across both Mediterranean and neighbouring/non-Mediterranean regions and in both men and women, although the magnitude of the association was somewhat stronger among participants from neighbouring/non-Mediterranean countries.

Within the MEDIET4ALL dataset, this interpretation is also consistent with previous findings showing that adherence to the Mediterranean lifestyle varies substantially across sex, region, and country [28,29,33], and that it is shaped by a multidimensional set of demographic, behavioural, and psychosocial factors rather than dietary factors alone [31]. The current study extends those findings by showing that this multidimensional lifestyle pattern is also associated with better subjective life satisfaction. However, the direction of this association cannot be determined from the present data. Although stronger adherence to healthy dietary and lifestyle practices may be associated with more favourable psychological functioning, the reverse explanation is also plausible: individuals with greater life satisfaction or lower psychological distress may have greater motivation, self-regulatory capacity, and resources to maintain healthier dietary, movement-related, sleep-related, and social behaviours. Prospective evidence has similarly indicated that relationships between diet quality, broader lifestyle behaviours, and mental health may be bidirectional rather thanoperating exclusively from lifestyle to psychological outcomes [6,44]. Nevertheless, the standardized association was modest, and the observational MEDLIFE score should not be interpreted as evidence that greater adherence causes improved life satisfaction. Moreover, the fully adjusted model explained approximately 17% of the variance in life satisfaction, indicating that life satisfaction is influenced by numerous additional determinants beyond those measured in the present survey. Personality traits, socioeconomic circumstances, interpersonal relationships, occupational factors, major life events, and other contextual influences are also likely to contribute substantially to interindividual differences in life satisfaction and should be considered in future research [45,46]. Furthermore, MEDLIFE components may not carry exactly equivalent behavioural or cultural meanings across all participating settings. Accordingly, the observed associations should be interpreted as relating to reported adherence to the behaviours represented by the instrument rather than to a universally homogeneous Mediterranean lifestyle construct.

### 4.2. Movement Behaviours and Social Participation Related-Correlates of Life Satisfaction and Psychological Distress

Higher physical activity was consistently associated with lower depression, anxiety, and stress, consistent with recent evidence supporting the mental-health benefits of physical activity [47]. However, because physical activity was assessed using the self-reported International Physical Activity Questionnaire–Short Form (IPAQ-SF), the observed associations should be interpreted cautiously. Although the IPAQ-SF is a widely validated instrument for population surveillance [38], self-reported physical activity remains susceptible to recall error and social desirability bias and may overestimate activity levels compared with objective measurements [48,49]. Furthermore, symptoms of depression or anxiety may influence both actual physical activity participation and participants’ perception and reporting of their habitual activity, making reverse directionality and mood-related reporting differences plausible explanations for part of the observed associations [6,50]. Interestingly, and contrary to the prevailing literature, higher physical activity expenditure was independently associated with lower life satisfaction in the fully adjusted model. This unexpected finding should not be interpreted as evidence that physical activity adversely affects life satisfaction. The IPAQ-SF estimates total activity-related energy expenditure and does not distinguish between leisure-time, occupational, transport-related, or domestic physical activity [38]. Consequently, higher MET values may partly reflect physically demanding or obligatory activities rather than discretionary exercise, which has been more consistently associated with improved psychological well-being [19,47]. Likewise, because the IPAQ-SF captures total weekly physical activity irrespective of its purpose or domain, higher reported activity levels may reflect occupational demands, active transportation, domestic activities, rehabilitation, or other health-related activities rather than discretionary leisure-time exercise, which has generally shown more consistent associations with psychological well-being [38,51,52].

The observed indirect associations involving physical activity and life satisfaction were statistically significant but very small, suggesting that these findings should be interpreted cautiously as modest, exploratory, and hypothesis-generating. Sedentary behaviour, in contrast, showed outcome-specific adverse associations, being positively related to depressive symptoms and stress. This distinction aligns with growing evidence recognising sedentary behaviour as an independent behavioural risk factor for adverse health outcomes [53,54]. Importantly, several large epidemiological studies have shown that prolonged sitting time may attenuate or even offset some of the beneficial effects of meeting recommended levels of physical activity [20,55,56]. These findings are also consistent with previous MEDIET4ALL analyses indicating that physical activity and sedentary behaviour should be considered distinct, although interrelated, lifestyle domains, as countries may exhibit favourable patterns in one domain but not necessarily the other [33], while sedentary behaviour showed stronger associations with poorer self-reported health than physical activity [32]. Taken together, the present findings reinforce the importance of considering these behaviours jointly rather than interchangeably.

Social participation likewise emerged as an important psychosocial correlate, showing a positive association with life satisfaction and an inverse association with depressive symptoms. This pattern aligns with previous MEDIET4ALL analyses showing that social participation is one of the most variable lifestyle dimensions across countries and may reflect broader sociocultural environments that support or constrain healthy behaviours and well-being [33]. It is also consistent with broader international evidence linking social participation and engagement with better well-being and lower depressive burden (Das et al., 2025; [23,57]. However, because the present analyses were cross-sectional, the relationship may be reciprocal rather than unidirectional. While social participation may provide social support, belonging, and opportunities that contribute to well-being, individuals with greater life satisfaction and lower psychological distress may also be more likely to initiate and maintain social engagement over time [58,59]. Accordingly, the observed indirect statistical associations involving life satisfaction cannot establish whether greater social participation contributes to improved psychological health, whether more favourable psychological functioning facilitates social participation, or whether both processes operate simultaneously over time. These findings suggest that Mediterranean lifestyle promotion should consider social environments and opportunities for collective engagement alongside individual dietary advice [30].

### 4.3. Sleep-Related Correlates of Life Satisfaction and Psychological Distress

Among the additional behavioural domains examined, sleep-related variables, particularly insomnia severity, showed the most consistent associations across both life satisfaction and psychological distress outcomes. Higher insomnia severity was inversely associated with life satisfaction and positively associated with depression, anxiety, and stress, while subjective sleep quality was positively associated with life satisfaction. These results are consistent with a large body of evidence indicating that sleep problems are closely linked to both reduced well-being and elevated mental health symptoms [60,61,62,63,64]. Meta-analytic evidence from randomized controlled trials has shown that improving sleep quality leads to meaningful reductions in depression, anxiety, and stress, supporting the view that sleep is not only a correlate of distress but also a potentially modifiable behavioural target for mental health promotion [21]. More recent evidence continues to support the close relationship between insomnia symptoms and common mental health problems across adult populations, with numerous longitudinal and meta-analytic studies reporting strong associations between insomnia and depression, anxiety, and psychological distress [21,35,65,66].

Importantly, this relationship is not necessarily unidirectional. Longitudinal and systematic-review evidence indicates that insomnia and other sleep disturbances may both precede and result from depression and anxiety [35,67,68,69]. Symptoms of depression and anxiety may disrupt sleep initiation and maintenance, but they may also influence individuals’ subjective appraisal and reporting of sleep quality, latency, and insomnia burden. Because both sleep and psychological outcomes were self-reported at the same assessment, the strong associations observed here may therefore reflect bidirectional relationships, overlapping symptom perception, or shared negative affectivity in addition to any underlying behavioural relationship.

The present findings complement this literature by suggesting that sleep may represent an important behavioural domain within the broader relationship between lifestyle patterns and psychological outcomes. In the current study, insomnia severity showed significant indirect associations within the observed associations between Mediterranean lifestyle adherence and both life satisfaction and psychological distress outcomes, indicating that the association between Mediterranean lifestyle adherence and psychological outcomes may be partly reflected in sleep-related characteristics. This interpretation is consistent with recent systematic reviews showing that stronger adherence to Mediterranean dietary and lifestyle patterns is generally associated with better sleep quality and more favourable sleep features, including lower insomnia burden [22,70]. Within the MEDIET4ALL framework, this is also coherent with previous determinant analyses showing that Mediterranean lifestyle adherence as well as health status in mediterranean and neighbour countries residents clusters with several interrelated behavioural and psychosocial domains, including sleep-related indicators and self-reported health status [30,32]. Taken together, these findings support the relevance of sleep health as one important behavioural domain within the broader Mediterranean lifestyle–mental health framework, although the interdependence among sleep indicators and the cross-sectional design preclude stronger mechanistic conclusions.

Sleep-related variables were also relevant to positive psychological functioning. Both insomnia severity and subjective sleep quality were associated with life satisfaction, supporting the view that sleep health relates not only to psychological distress but also to subjective positive psychological outcome. This is consistent with frameworks distinguishing positive well-being and psychological distress as related but non-equivalent dimensions of mental health [2,71].

### 4.4. Sociodemographic, Health-Related, and Regional Correlates

Several sociodemographic and health-related variables also showed meaningful associations. Better self-reported health status was consistently associated with both higher life satisfaction and lower psychological distress, which is consistent with recent regression analyses focusing on health status as a primary outcome and demonstrating strong associations with life satisfaction and psychological distress indicators such as anxiety [32]. Although these findings are not unexpected, they remain important because they indicate that subjective health perception remains a key correlate of life satisfaction even after accounting for multiple lifestyle domains. Higher education and marital or cohabiting status were associated with more favourable psychological outcomes, possibly reflecting differences in health literacy, coping resources, companionship, and social support [72,73,74]. Older age was associated with lower distress, although this interpretation should remain cautious given the cross-sectional design [75,76]. Alcohol consumption was positively associated with all distress dimensions, consistent with literature showing co-occurrence between alcohol-related behaviours and common mental health symptoms [77,78]. A particularly interesting result concerns the regional contrast. In the adjusted models, residing in Mediterranean countries was associated with lower life satisfaction and higher levels of psychological distress. This should not be interpreted as evidence against the Mediterranean lifestyle itself. Rather, these regional coefficients should be interpreted cautiously because geographical residence should not be considered a proxy for contemporary adherence to Mediterranean lifestyle practices, and the behavioural meaning of individual MEDLIFE components may not be fully equivalent across participating cultural and linguistic settings. Regional coefficients should therefore be interpreted as broad contextual associations and may reflect differences in healthcare systems, socioeconomic conditions, cultural norms, dietary practices, social structures, environmental characteristics, geopolitical circumstances, and other contextual factors that were not fully measured in the present study [6,11,79], as well as cross-country differences in recruitment channels and participant composition. Accordingly, these coefficients should not be interpreted as evidence of an effect attributable specifically to residence in a Mediterranean country. Although the magnitude of the association between Mediterranean lifestyle adherence and life satisfaction differed somewhat between the two broad regional groupings, the positive association remained evident in both groups in the region-specific sensitivity analyses, supporting the robustness of the principal finding while also indicating that regional context may modify its strength. Previous MEDIET4ALL studies already showed substantial heterogeneity across Mediterranean and non-Mediterranean countries in adherence profiles, psychosocial factors, movement behaviours, and social participation, indicating that modern sociocultural, economic, and environmental contexts may substantially modify lifestyle patterns and their health implications [29,32,33]. Therefore, the present regional findings arguably reinforce the need to distinguish between the Mediterranean lifestyle as a behavioural construct and the simple fact of living in a Mediterranean country.

### 4.5. Integrated Behavioural Framework and Public Health Implications

Taken together, the primary regression findings indicate that Mediterranean lifestyle adherence, movement behaviours, social participation, sleep characteristics, and health-related factors show distinct but interrelated associations with life satisfaction and psychological distress. The exploratory cross-sectional indirect-association analyses provide an additional hypothesis-generating description of how selected variables covary within this broader framework. Because reciprocal and alternative statistical orderings are equally plausible, these patterns should not be interpreted as establishing temporal sequence or causal direction. They instead provide hypotheses for future longitudinal and intervention-based studies. From a public-health perspective, the findings support integrated Mediterranean lifestyle promotion rather than isolated behavioural recommendations. Within this framework, sleep health appears to be an important behavioural domain, particularly because insomnia severity was consistently associated with distress and showed exploratory indirect associations with Mediterranean lifestyle adherence and psychological outcomes. Social participation and physical activity may likewise represent complementary behavioural domains to consider when designing interventions aimed at enhancing life satisfaction and resilience. This interpretation is consistent with broader frameworks emphasizing that mental health promotion requires integrated and context-sensitive public health approaches that address clusters of behavioural and psychosocial determinants rather than isolated habits [3,6]. Within the wider MEDIET4ALL programme, these findings support the development of culturally adapted, multidimensional interventions that combine dietary guidance with sleep hygiene, movement-promoting strategies, and opportunities for social engagement, while accounting for regional and contextual heterogeneity.

### 4.6. Strengths and Limitations

This study has several strengths. It used a large multinational sample spanning Mediterranean and neighbouring countries and relied on a standardized multilingual survey using internationally validated self-report instruments, which represents the standard methodological approach for large-scale multinational behavioural epidemiological studies. This enabled harmonized assessment of lifestyle, psychosocial, and health-related variables across participating countries while simultaneously examining both positive and negative dimensions of psychological functioning. The study also considered a broad set of behavioural, psychosocial, and sociodemographic correlates within one integrated hierarchical analytical framework, thereby extending previous MEDIET4ALL work from lifestyle adherence and health-status outcomes to psychological health as a primary outcome domain. The robustness of the principal findings was further supported through prespecified region- and sex-specific sensitivity analyses together with false-discovery-rate correction for multiple testing. Finally, the exploratory cross-sectional indirect-association analyses provided a useful conceptual extension by generating hypotheses regarding the statistical organization of the observed associations between lifestyle behaviours and psychological outcomes.

Several limitations should nevertheless be acknowledged. First, the cross-sectional design precludes causal inference and temporal ordering; accordingly, the observed associations, including the exploratory indirect statistical associations, should not be interpreted as evidence of causal mediation, temporal pathways, or behavioural mechanisms. Second, all measures were self-reported and may therefore be affected by recall and social desirability biases, response fatigue, and measurement error. Collection of predictors and outcomes from the same respondents at a single assessment also introduces potential common-method variance. This is particularly relevant to physical activity, sedentary behaviour, sleep indicators, and BMI derived from self-reported height and weight. Third, the non-probability online convenience and network-based recruitment strategy may have introduced selection bias by preferentially reaching individuals active online or interested in health-related topics. The sample was also relatively young, comparatively highly educated, and unevenly distributed across countries. Consequently, findings apply to the recruited multinational analytical sample and should not be interpreted as nationally representative estimates or population-level comparisons. Together with the pooled and complete-case analytical approach, these characteristics may also incompletely capture country-specific heterogeneity. Fourth, some cross-cultural measurement non-equivalence cannot be excluded. Although multilingual translation, harmonization, and pilot reliability procedures were implemented, formal measurement-invariance testing was not conducted for all instruments. Residual confounding by unmeasured socioeconomic, cultural, healthcare-system, environmental, geopolitical, and other contextual factors also remains possible, particularly for the broad Mediterranean versus neighbouring/non-Mediterranean comparison. Regional associations should therefore not be interpreted as effects attributable specifically to residence in a Mediterranean country. Finally, although false-discovery-rate correction supported most fully adjusted associations, the number of predictors and outcomes warrants caution when interpreting weaker or outcome-specific findings. These limitations highlight the need for longitudinal and intervention studies incorporating objective behavioural measures and formal cross-cultural validation.

## 5. Conclusions and Future Directions

In conclusion, within the recruited multinational MEDIET4ALL analytical sample, life satisfaction and psychological distress are associated with a multidimensional set of lifestyle, sleep-related, psychosocial, and sociodemographic factors within the MEDIET4ALL framework. Mediterranean lifestyle adherence, social participation, and physical activity were significantly associated with psychological health. Sleep-related factors, particularly insomnia severity and subjective sleep quality, emerged as the most consistent correlates across both life satisfaction and psychological distress outcomes. Exploratory indirect statistical associations were also observed for selected relationships involving insomnia severity and life satisfaction. These findings support the value of an integrated Mediterranean lifestyle perspective and suggest that future mental health promotion strategies may benefit from addressing sleep, movement, social participation, and lifestyle behaviours together rather than in isolation.

At the same time, given the cross-sectional design, these findings should be interpreted as statistical associations rather than causal relationships. Future research should prioritise longitudinal and intervention-based designs to determine whether these associations persist prospectively and to clarify their temporal relationships. In addition, expanding the analytical framework to include broader contextual and cultural determinants, such as sociocultural norms, dominant religious practices, social cohesion, and culturally embedded health behaviours, may further improve the understanding of lifestyle–well-being relationships across diverse populations. Finally, integrating these multidimensional behavioural and psychosocial profiles with long-term health indicators, such as morbidity trajectories and life expectancy, could provide a more comprehensive perspective on the public health relevance of Mediterranean lifestyle adherence.

## Figures and Tables

**Table 1:** Characteristics of the study population and distribution of key sociodemographic, lifestyle, sleep, and psychological variables in the MEDIET4ALL sample (*n* = 4010).

Variable	Total Sample
Age, years	37.25 ± 15.39
Female (%)	59.5%
Education level, (bachelor/master %)	58.8%
Employment status, (employed %)	50.7%
Marital status, (married %)	43.5%
Living environment, (urban %)	66.3%
BMI (kg/m^2^)	24.82 ± 4.85
Tabaco Smokers (%)	25%
Self-reported health status, (healthy %)	75.2%
MEDLIFE total score	14.01 ± 3.15
Physical activity (MET-min/week)	1768.22 ± 2176.93
Sitting time (h/day)	5.6 ± 3.04
Sleep duration (h/night)	6.82 ± 1.7
Sleep latency	30.4 ± 33.68
Sleep efficiency	93.15 ± 7.16
ISI score	9.67 ± 5.83
SSPQ score	37.91 ± 10.06
STuQL score	8.89 ± 2.74
MBQ score	6.87 ± 1.29
SLSQ score	14.24 ± 4.52
Depression score	13.37 ± 5.02
Anxiety score	12.33 ± 4.73
Stress score	14.16 ± 4.78

**Table 2:** Hierarchical multiple linear regression analyses examining associations between sociodemographic, health-related, lifestyle, and psychosocial factors and life satisfaction.

Predictor	M1 B	M2 B	M3 B	M4 B	M5 B	M6 B	M7 B (95% CI)	M7 β	M7 p
Age	−0.007	0.009	0.002	0.002	−0.008	0.005			
Sex	−0.263	−0.328	−0.323	−0.315	−0.066	0.005			
Region	−0.323	−0.478	−0.580	−0.580	−0.518	−0.466	−0.478 (−0.752; −0.205)	−0.051	0.001
Education	0.381	0.322	0.275	0.295	0.272	0.208	0.212 (0.112; 0.312)	0.061	0.000
Employment	0.015	0.022	0.029	0.025	0.048	0.007			
Marital status	0.394	0.514	0.562	0.557	0.594	0.563	0.609 (0.392; 0.826)	0.084	0.000
Living environment	−0.287	−0.256	−0.192	−0.206	−0.179	−0.118			
BMI		−0.072	−0.062	−0.058	−0.049	−0.045	−0.043 (−0.071; −0.015)	−0.046	0.002
Smoking		0.143	0.101	0.111	0.075	0.053			
Alcohol		0.020	−0.041	−0.041	0.151	−0.010			
Health status		1.111	1.029	1.002	0.643	0.618	0.611 (0.385; 0.838)	0.081	0.000
MEDLIFE total			0.241	0.230	0.176	0.120	0.129 (0.086; 0.172)	0.09	0.000
IPAQ (MET)				0.000	0.000	0.000	−0.074 (−0.116; −0.031)	−0.05	0.001
Sitting time				−0.101	−0.084	−0.071			
Sleep duration					0.026	−0.005			
Sleep latency					−0.017	−0.015			
Sleep efficiency					−0.046	−0.036			
Subjective sleep quality					0.713	0.589	0.598 (0.398; 0.797)	0.104	0.000
ISI total score					−0.120	−0.133	−0.149 (−0.175; −0.122)	−0.192	0.000
SSPQ total score						0.081	0.079 (0.065; 0.093)	0.176	0.000
STuQL total score						0.031			
**Number of predictors (k)**	7	11	12	14	19	21	10		
**R^2^**	0.019	0.048	0.075	0.08	0.146	0.172	0.168		
**Adjusted R^2^**	0.017	0.045	0.072	0.077	0.142	0.167	0.166		
**ΔR^2^**	0.019	0.029	0.027	0.005	0.066	0.026	0.168		
**F change**	9.86	30.41	116.83	10.12	62.14	61.59	80.58		

B = unstandardized regression coefficient; CI = confidence interval; β = standardized beta coefficient; Adjusted R^2^ = adjusted R-squared / adjusted coefficient of determination; F = F-statistic for the overall regression model; *p* for model = overall model *p*-value; IPAQ score = International Physical Activity Questionnaire score; ISI = Insomnia Severity Index; SSPQ-L = Short Social Participation Questionnaire–Lockdowns.

**Table 3:** Auxiliary parsimonious Model 7 summaries of factors associated with depression, anxiety, and stress.

Predictor	Depression B (95% CI)	β	*p*	Anxiety B (95% CI)	β	*p*	Stress B (95% CI)	β	*p*
Age (Years)	−0.039 (−0.05; −0.027)	−0.118	0.000	−0.039 (−0.048; −0.03)	−0.13	0.000	−0.042 (−0.051; −0.033)	−0.14	0.000
Gender				0.357 (0.093; 0.62)	0.04	0.008	0.777 (0.505; 1.048)	0.08	0.000
Region	0.549 (0.264; 0.833)	0.052	0.000	0.568 (0.302; 0.834)	0.06	0.000	0.752 (0.478; 1.026)	0.08	0.000
Education	−0.137 (−0.241; −0.032)	−0.036	0.000	−0.188 (−0.284; −0.092)	−0.05	0.000			
Employment							−0.095 (−0.202; 0.012)	−0.02	0.082
Marital status	−0.293 (−0.553; −0.033)	−0.036	0.010						
Alcohol	1.116 (0.889; 1.344)	0.133	0.027	1.148 (0.933; 1.363)	0.15	0.000	0.955 (0.736; 1.175)	0.12	0.000
Health	−0.728 (−0.966; −0.49)	−0.087	0.000	−0.766 (−0.987; −0.546)	−0.10	0.000	−0.571 (−0.798; −0.344)	−0.07	0.000
IPAQ score	0 (0; 0)	−0.047	0.000	0 (0; 0)	−0.08	0.000	0 (0; 0)	−0.03	0.024
Sitting	0.069 (0.025; 0.113)	0.042	0.001				0.058 (0.016; 0.1)	0.04	0.007
Number of hours of sleep				0.151 (0.076; 0.227)	0.05	0.000			
Falling asleep				0.016 (0.012; 0.02)	0.11	0.000			
Sleep quality	0.47 (0.262; 0.677)	0.073	0.002	0.603 (0.407; 0.799)	0.10	0.000	0.182 (−0.016; 0.379)	0.03	0.071
ISI	0.406 (0.377; 0.434)	0.471	0.000	0.345 (0.317; 0.373)	0.43	0.000	0.36 (0.333; 0.387)	0.44	0.000
SSPQ-L	−0.032 (−0.046; −0.018)	−0.064	0.000	0.03 (0.017; 0.043)	0.06	0.000			
**Predictors, k**	11			12			10		
**Adjusted R^2^**	0.272			0.294			0.262		
**F**	137.43			140.24			143.43		
***p* for model**	0.000			0.000			0.000		

B = unstandardized regression coefficient; CI = confidence interval; β = standardized beta coefficient; Adjusted R^2^ = adjusted R-squared / adjusted coefficient of determination; F = F-statistic for the overall regression model; *p* for model = overall model *p*-value; IPAQ score = International Physical Activity Questionnaire score; ISI = Insomnia Severity Index; SSPQ-L = Short Social Participation Questionnaire–Lockdowns. **Note:** Results are derived from the parsimonious Model 7, which retained predictors selected from the fully adjusted Model 6. Inferential interpretation was based primarily on Model 6.

**Table 4:** Results of the exploratory bootstrapped cross-sectional indirect-association analyses examining statistical associations between Mediterranean lifestyle adherence, behavioural factors, and psychological outcomes in the MEDIET4ALL sample.

Statistical Model Specification		Direct β	Indirect β	95% CI
MedLife–insomnia conceptual model	A: MEDLIFE → ISI → SLSQ	0.2034	0.0374	0.0262; 0.0496
	B1: MEDLIFE → ISI → Depression	−0.0590	−0.0727	−0.0943; −0.0528
	B2: MEDLIFE → ISI → Anxiety	0.0060	−0.0650	−0.0838; −0.0465
	B3: MEDLIFE → ISI → Stress	−0.0280	−0.0688	−0.0895; −0.0495
Physical activity–life satisfaction conceptual model	C1: Physical activity → SLSQ → Depression	−0.0001	0.0000	−0.0001; 0.0000
	C2: Physical activity → SLSQ → Anxiety	−0.0002	0.0000	0.0000; 0.0000
	C3: Physical activity → SLSQ → Stress	−0.0001	0.0000	0.0000; 0.0000
Social participation–life satisfaction conceptual model	D2: Social participation → SLSQ → Anxiety	0.0553	−0.0240	−0.0288; −0.0193
	D3: Social participation → SLSQ → Stress	0.0282	−0.0315	−0.0375; −0.0259

β = Beta coefficient; CI = Confidence interval; MEDLIFE = Mediterranean Lifestyle Index; ISI = Insomnia Severity Index; SLSQ = Short Life Satisfaction Questionnaire. **Notes: 1.** Values reported as 0.0000 represent very small non-zero estimates that round to zero at four decimal places. Statistical significance was assessed using the unrounded bootstrapped estimates and 95% confidence intervals **2.** These exploratory bootstrapped indirect-association analyses are based on cross-sectional data and are intended to quantify statistical associations within the proposed conceptual framework. They should not be interpreted as evidence of temporal ordering, causal direction, or mediation.

## Data Availability

Public deposition of the MEDIET4ALL dataset is currently restricted because the database contains sensitive information collected across multiple countries and remains governed by ongoing consortium analyses, ethical requirements, data-protection obligations, and project agreements. Requests for access to de-identified data or relevant analytical materials may be directed to the corresponding author and will be considered on a reasonable basis, subject to applicable ethical approval, GDPR provisions, and authorization under the MEDIET4ALL consortium agreement.

## Supplementary Materials

The following supporting information can be downloaded at https://www.mdpi.com/article/10.3390/nu18162708/s1, Table S1. Hierarchical multiple linear regression analyses examining associations between sociodemographic, health-related, lifestyle, and psychosocial factors and Depression; Table S2. Hierarchical multiple linear regression analyses examining associations between sociodemographic, health-related, lifestyle, and psychosocial factors and Anxiety; Table S3. Hierarchical multiple linear regression analyses examining associations between sociodemographic, health-related, lifestyle, and psychosocial factors and Stress; Table S4. Collinearity diagnostics for predictors included in the fully adjusted model examining correlates of SLSQ; Table S5. Sensitivity analysis examining whether the association between Mediterranean lifestyle adherence and life satisfaction differed according to Mediterranean versus neighbouring/non-Mediterranean region; Table S6. Sensitivity analysis examining whether the association between Mediterranean lifestyle adherence and life satisfaction differed according to sex; Table S7. Nominal and Benjamini–Hochberg false-discovery-rate-adjusted results for nominally significant predictors in the fully adjusted psychological-outcome models (model 6).
