# Supplementary material for: Life Satisfaction and Psychological Distress in Relation to Mediterranean Lifestyle Adherence and Behavioural Correlates: Findings from the MEDIET4ALL International Survey
Source: Nutrients. 2026 Aug 19;18(16):2708. doi: 10.3390/nu18162708 (PMC13516983; doi:10.3390/nu18162708)
Supplement: Supplementary file 1 [file nutrients-18-02708-s001.zip › nutrients-4460826-supplementary.pdf]

Supplementary Material

Table S1. Hierarchical multiple linear regression analyses examining associations between sociodemographic, health-related, lifestyle, and psychosocial factors and Depression

Predictor	M1 β	M2 β	M3 β	M4 β	M5 β	M6 β
Age	-0.17	-0.20	-0.19	-0.19	-0.09	-0.11
Sex	0.04	0.08	0.08	0.07	0.03	0.02
Region	0.03	0.03	0.04	0.04	0.06	0.06
Education	-0.08	-0.05	-0.05	-0.05	-0.04	-0.03
Employment	0.01	0.01	0.01	0.01	-0.01	0.00
Marital status	0.00	-0.03	-0.03	-0.03	-0.04	-0.04
Living environment	0.02	0.00	0.00	0.00	0.00	0.00
BMI		0.04	0.04	0.03	0.01	0.01
Smoking		-0.03	-0.03	-0.03	-0.02	-0.02
Alcohol		0.17	0.18	0.18	0.13	0.13
Health status		-0.17	-0.16	-0.16	-0.09	-0.08
MEDLIFE total			-0.08	-0.07	-0.03	-0.02
IPAQ (MET)				-0.05	-0.05	-0.04
Sitting time				0.05	0.04	0.04
Sleep duration					0.03	0.04
Sleep latency					0.07	0.07
Sleep efficiency					0.00	0.00
Subjective sleep quality					0.07	0.08
ISI total score					0.44	0.45
SSPQ total score						-0.06
STuQL total score						0.01
**Number of predictors (k)**	7	11	12	14	19	21
**R^2**	0.033	0.097	0.103	0.109	0.278	0.281
**Adjusted R^2**	0.031	0.094	0.101	0.105	0.275	0.277
**ΔR^2**	0.033	0.064	0.007	0.005	0.17	0.003
**F change**	19.29	70.73	29.98	11.82	187.61	8.19

Table S2. Hierarchical multiple linear regression analyses examining associations between sociodemographic, health-related, lifestyle, and psychosocial factors and Anxiety.

**Predictor**	**M1 β**	**M2 β**	**M3 β**	**M4 β**	**M5 β**	**M6 β**
Age	-0.23	-0.25	-0.25	-0.25	-0.15	-0.14
Sex	0.05	0.09	0.09	0.08	0.03	0.04
Region	0.03	0.03	0.04	0.03	0.06	0.06
Education	-0.07	-0.06	-0.05	-0.06	-0.04	-0.05
Employment	0.04	0.03	0.03	0.03	0.01	0.01
Marital status	0.05	0.02	0.02	0.02	0.01	0.01
Living environment	0.02	0.01	0.01	0.01	0.01	0.01
BMI		0.03	0.03	0.02	0.00	0.00
Smoking		-0.01	-0.01	0.00	0.01	0.00
Alcohol		0.20	0.20	0.21	0.15	0.15
Health status		-0.16	-0.16	-0.16	-0.09	-0.10
MEDLIFE total			-0.04	-0.03	0.01	0.00
IPAQ (MET)				-0.08	-0.08	-0.08
Sitting time				0.01	0.00	0.00
Sleep duration					0.05	0.04
Sleep latency					0.14	0.14
Sleep efficiency					0.03	0.04
Subjective sleep quality					0.11	0.10
ISI total score					0.43	0.43
SSPQ total score						0.07
STuQL total score						-0.02
**Number of predictors (k)**	7	11	12	14	19	21
**R^2**	0.048	0.119	0.12	0.126	0.293	0.297
**Adjusted R^2**	0.046	0.117	0.118	0.123	0.29	0.293
**ΔR^2**	0.048	0.071	0.001	0.006	0.167	0.004
**F change**	28.76	80.6	6.43	13.77	188.5	10.49

Table S3. Hierarchical multiple linear regression analyses examining associations between sociodemographic, health-related, lifestyle, and psychosocial factors and Stress

Predictor	M1 β	M2 β	M3 β	M4 β	M5 β	M6 β
**Age**	-0.21	-0.23	-0.23	-0.23	-0.14	-0.14
**Sex**	0.09	0.13	0.13	0.12	0.08	0.08
**Region**	0.05	0.06	0.06	0.06	0.07	0.07
**Education**	-0.04	-0.02	-0.02	-0.02	-0.01	-0.01
**Employment**	-0.01	-0.02	-0.02	-0.02	-0.03	-0.03
**Marital status**	0.04	0.02	0.02	0.02	0.01	0.01
**Living environment**	0.01	0.00	0.00	0.00	0.00	0.00
**BMI**		0.03	0.02	0.02	0.00	0.00
**Smoking**		-0.01	-0.01	-0.01	0.00	0.00
**Alcohol**		0.16	0.17	0.17	0.12	0.12
**Health status**		-0.15	-0.15	-0.14	-0.07	-0.07
**MEDLIFE total**			-0.06	-0.06	-0.01	-0.01
**IPAQ (MET)**				-0.03	-0.03	-0.03
**Sitting time**				0.05	0.04	0.04
**Sleep duration**					-0.01	-0.01
**Sleep latency**					0.10	0.10
**Sleep efficiency**					0.05	0.05
**Subjective sleep quality**					0.04	0.04
**ISI total score**					0.42	0.42
**SSPQ total score**						0.00
**STuQL total score**						-0.01
**Number of predictors (k)**	7	11	12	14	19	21
**R^2**	0.045	0.097	0.101	0.104	0.267	0.267
**Adjusted R^2**	0.043	0.095	0.098	0.101	0.263	0.263
**ΔR^2**	0.045	0.052	0.004	0.003	0.163	0
**F change**	26.8	58.04	17.24	7.31	176.9	0.24

Table S4. Collinearity diagnostics for predictors included in the fully adjusted model examining correlates of SLSQ

Predictor	Tolerance	VIF
Age	,542	1,844
Sex	,871	1,149
Region	,910	1,098
Education	,902	1,109
Employment	,913	1,095
Marital status	,650	1,538
Living environment	,941	1,063
BMI	,854	1,171
Smoking	,924	1,083
Alcohol	,893	1,119
Health status	,839	1,193
MEDLIFE total	,869	1,150
IPAQ (MET)	,927	1,078
Sitting time	,974	1,027
Sleep duration	,414	2,413
Sleep latency	,065	15,393
Sleep efficiency	,060	16,679
Subjective sleep quality	,634	1,576
ISI total score	,579	1,727
SSPQ total score	,798	1,254
STuQL total score	,991	1,009

VIF: variance inflation factor. Tolerance = 1/VIF. Collinearity diagnostics were derived from the fully adjusted regression model (Model 6).

Table S5. Sensitivity analysis examining whether the association between Mediterranean lifestyle adherence and life satisfaction differed according to Mediterranean versus neighbouring/non-Mediterranean region

Region	n	B	β	SE	p	95% CI
Neighbouring/non-Mediterranean	1416	0.195	0.138	0.036	<0.001	0.124 to 0.267
Mediterranean	2594	0.085	0.059	0.028	0.002	0.030 to 0.140
Interaction analysis (fully adjusted pooled model)						
**Interaction term**		**B**	**β**	**SE**	**p**	**95% CI**
MEDLIFE × Region		-0.110	-0.040	0.044	0.011	-0.196 to -0.025

B = unstandardized regression coefficient; β = standardized beta coefficient; CI = confidence interval; SE = standard error. Results are derived from the fully adjusted hierarchical regression model used for the primary life satisfaction analyses. Subgroup models were adjusted for all covariates included in the fully adjusted model except the corresponding stratification variable. MEDLIFE scores were mean-centred before construction of the interaction term. Formal effect modification was evaluated using the fully adjusted pooled interaction model. The association between MEDLIFE adherence and life satisfaction was observed in both regional groups; however, the significant interaction term indicated that the magnitude of the association was stronger among participants from neighbouring/non-Mediterranean countries.

Table S6. Sensitivity analysis examining whether the association between Mediterranean lifestyle adherence and life satisfaction differed according to sex

Sex	n	B	β	SE	p	95% CI
Men	1625	0.097	0.069	0.034	0.004	0.030 to 0.164
Women	2385	0.134	0.092	0.029	<0.001	0.076 to 0.191
Interaction analysis (fully adjusted pooled model)						
**Interaction term**		**B**	**β**	**SE**	**p**	**95% CI**
MEDLIFE × Sex		0.016	0.006	0.042	0.711	-0.067 to 0.098

B = unstandardized regression coefficient; **β** = standardized beta coefficient; **CI** = confidence interval; **SE** = standard error. Results are derived from the fully adjusted hierarchical regression model used for the primary life satisfaction analyses. Subgroup models were adjusted for all covariates included in the fully adjusted model except the corresponding stratification variable. MEDLIFE scores were mean-centred before construction of the interaction term. Formal effect modification was evaluated using the fully adjusted pooled interaction model. The association between MEDLIFE adherence and life satisfaction was observed in both men and women. The non-significant interaction term indicated no evidence that the magnitude of the association differed between sexes.

Table S7. Nominal and Benjamini–Hochberg false-discovery-rate-adjusted results for nominally significant predictors in the fully adjusted psychological-outcome models (model 6).

Outcome	Predictor	B (95% CI)	β	Nominal p	FDR q	Retained after FDR
**Life satisfaction (Primary)**	Region	−0.466 (−0.745 to −0.186)	−0.049	0.0011	0.0026	Yes
	Education	0.208 (0.105 to 0.311)	0.060	<0.001	<0.001	Yes
	Marital status	0.563 (0.309 to 0.817)	0.078	<0.001	<0.001	Yes
	BMI	−0.045 (−0.074 to −0.017)	−0.049	0.0018	0.0038	Yes
	Self-reported health status	0.618 (0.386 to 0.850)	0.082	<0.001	<0.001	Yes
	MEDLIFE total score	0.120 (0.077 to 0.164)	0.084	<0.001	<0.001	Yes
	Sitting time	−0.071 (−0.114 to −0.029)	−0.048	0.0010	0.0026	Yes
	Subjective sleep quality	0.589 (0.385 to 0.793)	0.102	<0.001	<0.001	Yes
	ISI total score	−0.133 (−0.162 to −0.104)	−0.172	<0.001	<0.001	Yes
	SSPQ total score	0.081 (0.066 to 0.095)	0.180	<0.001	<0.001	Yes
**Depression (Secondary)**	Age	−0.035 (−0.046 to −0.023)	−0.107	<0.001	<0.001	Yes
	Region	0.587 (0.297 to 0.876)	0.056	<0.001	<0.001	Yes
	Education	−0.128 (−0.235 to −0.022)	−0.033	0.0181	0.0346	Yes
	Marital status	−0.324 (−0.587 to −0.061)	−0.040	0.0157	0.0330	Yes
	Alcohol consumption	1.112 (0.879 to 1.346)	0.133	<0.001	<0.001	Yes
	Self-reported health status	−0.705 (−0.945 to −0.465)	−0.084	<0.001	<0.001	Yes
	IPAQ score	−0.00011 (−0.00017 to −0.00004)	−0.046	0.0011	0.0029	Yes
	Sitting time	0.063 (0.019 to 0.107)	0.038	0.0051	0.0119	Yes

Outcome	Predictor	B (95% CI)	β	Nominal p	FDR q	Retained after FDR
	Subjective sleep quality	0.487 (0.276 to 0.699)	0.076	<0.001	<0.001	Yes
	ISI total score	0.385 (0.355 to 0.415)	0.447	<0.001	<0.001	Yes
	SSPQ total score	−0.030 (−0.044 to −0.015)	−0.060	<0.001	<0.001	Yes
**Anxiety (Secondary)**	Age	−0.041 (−0.052 to −0.031)	−0.135	<0.001	<0.001	Yes
	Sex	0.357 (0.088 to 0.626)	0.037	0.0092	0.0176	Yes
	Region	0.576 (0.306 to 0.846)	0.058	<0.001	<0.001	Yes
	Education	−0.180 (−0.280 to −0.081)	−0.050	<0.001	<0.001	Yes
	Alcohol consumption	1.150 (0.933 to 1.367)	0.146	<0.001	<0.001	Yes
	Self-reported health status	−0.754 (−0.978 to −0.530)	−0.096	<0.001	<0.001	Yes
	IPAQ score	−0.00017 (−0.00023 to −0.00012)	−0.080	<0.001	<0.001	Yes
	Sleep duration	0.120 (0.007 to 0.233)	0.043	0.0367	0.0643	**No**
	Sleep latency	0.020 (0.006 to 0.034)	0.143	0.0061	0.0127	Yes
	Subjective sleep quality	0.605 (0.408 to 0.802)	0.100	<0.001	<0.001	Yes
	ISI total score	0.345 (0.317 to 0.373)	0.425	<0.001	<0.001	Yes
	SSPQ total score	0.031 (0.017 to 0.045)	0.065	<0.001	<0.001	Yes
**Stress (Secondary)**	Age	−0.044 (−0.055 to −0.032)	−0.141	<0.001	<0.001	Yes
	Sex	0.752 (0.475 to 1.029)	0.077	<0.001	<0.001	Yes
	Region	0.736 (0.458 to 1.015)	0.074	<0.001	<0.001	Yes
	Employment status	−0.112 (−0.223 to −0.0003)	−0.028	0.0495	0.1039	**No**

Outcome	Predictor	B (95% CI)	β	Nominal p	FDR q	Retained after FDR
	Alcohol consumption	0.943 (0.719 to 1.167)	0.118	<0.001	<0.001	Yes
	Self-reported health status	−0.555 (−0.786 to −0.325)	−0.070	<0.001	<0.001	Yes
	IPAQ score	−0.00007 (−0.00014 to −0.00001)	−0.034	0.0157	0.0412	Yes
	Sitting time	0.055 (0.012 to 0.097)	0.035	0.0112	0.0336	Yes
	Subjective sleep quality	0.217 (0.014 to 0.420)	0.036	0.0361	0.0842	**No**
	ISI total score	0.346 (0.317 to 0.374)	0.422	<0.001	<0.001	Yes

B = unstandardized regression coefficient; CI = confidence interval; β = standardized beta coefficient; FDR = false discovery rate; ISI = Insomnia Severity Index; IPAQ = International Physical Activity Questionnaire; MEDLIFE = Mediterranean Lifestyle Index; SSPQ = Short Social Participation Questionnaire. Benjamini–Hochberg correction was applied separately to the 21 predictor tests within each fully adjusted outcome model (Model 6). Life satisfaction was the primary outcome, whereas depression, anxiety, and stress were secondary outcomes and were treated as separate multiplicity families. The intercept was excluded from multiplicity correction. FDR-adjusted statistical significance was defined as $q < 0.05$. Only predictors with nominal $p < 0.05$ are displayed; complete Model 6 results are available in Table 2 and Supplementary Tables S1–S3.
